# Developing selective media for quantification of multispecies biofilms following antibiotic treatment

**DOI:** 10.1371/journal.pone.0187540

**Published:** 2017-11-09

**Authors:** Eva Vandeplassche, Tom Coenye, Aurélie Crabbé

**Affiliations:** Laboratory of Pharmaceutical Microbiology, Ghent University, Ghent, Belgium; Institute for Bioengineering of Catalonia (IBEC), SPAIN

## Abstract

The lungs of cystic fibrosis (CF) patients are chronically colonized by a polymicrobial biofilm community, leading to difficult-to-treat infections. To combat these infections, CF patients are commonly treated with a variety of antibiotics. Understanding the dynamics of polymicrobial community composition in response to antibiotic therapy is essential in the search for novel therapies. Culture-dependent quantification of individual bacteria from defined multispecies biofilms is frequently carried out by plating on selective media. However, the influence of the selective agents in these media on quantitative recovery before or after antibiotic treatment is often unknown. In the present study we developed selective media for six bacterial species that are frequently co-isolated from the CF lung, i.e. *Pseudomonas aeruginosa*, *Staphylococcus aureus*, *Streptococcus anginosus*, *Achromobacter xylosoxidans*, *Rothia mucilaginosa*, and *Gemella haemolysans*. We show that certain supplementations to selective media strongly influence quantitative recovery of (un)treated biofilms. Hence, the developed media were optimized for selectivity and quantitative recovery before or after treatment with antibiotics of four major classes, i.e. ceftazidime, ciprofloxacin, colistin, or tobramycin. Finally, in a proof of concept experiment the novel selective media were applied to determine the community composition of multispecies biofilms before and after treatment with tobramycin.

## Introduction

Chronic respiratory tract infections are the main cause of morbidity and mortality in cystic fibrosis (CF) patients [1]. In the CF lung environment, a polymicrobial community is the source of these continuing infections, which results in an inflammatory response and eventually leads to lung deterioration [2–5]. Treatment of chronic lung infections in CF patients is particularly challenging as members of the bacterial community form biofilms [6] which are highly resistant to antimicrobial therapy [7,8]. The presence of biofilm-related infections has a significant impact on clinical health outcomes, more specifically on lung function [9].

Interspecies interactions in polymicrobial communities have been shown to influence various phenotypic characteristics of individual bacteria, such as antimicrobial susceptibility and production of virulence factors [10]. Hence, the importance of studying the role of complex bacterial communities in disease progression and antibiotic susceptibility is generally acknowledged [10,11]. To further elucidate the role of interspecies interactions in antibiotic susceptibility of defined polymicrobial communities, *in vitro* studies have provided valuable insights [12–14]. To this end, quantification of bacteria in multispecies consortia before and after antibiotic treatment is performed using culture-dependent and -independent techniques. One of the most commonly used culture-dependent quantification methods for biofilms is determination of CFU (colony forming unit) counts on selective media. Most bacteria in the CF lung are culturable, making this an effective approach for quantification of CF-related biofilms [15,16]. While selective media are available for a broad range of microorganisms, many are focused solely on isolation from clinical or environmental samples and not on quantitative recovery [17–20]. Few studies have investigated how the quantitative recovery on selective media compares to that on a general medium [21–23], although this is essential to obtain an accurate picture of the amounts of particular taxa in bacterial communities. Furthermore, the influence of antibiotic treatment on the recovery on a selective medium, which is often supplemented with various antimicrobial substances, has not yet been investigated. This is important, as prior antibiotic exposure could increase susceptibility to the selective agent(s) in the medium, and therefore affect quantitative recovery.

The present study aims at developing selective media for six bacterial species commonly found together in the CF lung in order to allow accurate quantification of individual species in complex biofilm communities, before and after antibiotic treatment. Furthermore, previously described selective media were evaluated in the process as well. Our approach for the development of a selective medium for each of the species of interest consists of three steps. First, the selectivity for each species was assessed using planktonic cultures. Second, the quantitative recovery of single-species biofilms was compared between different media. Third, the single-species biofilms were treated with antibiotics of four different classes after which the recovery was evaluated again. Based on results obtained in each step, media composition was optimized to improve quantitative recovery on the selective medium. Finally, the developed media were tested in various experiments, to confirm their applicability for quantification of bacteria from planktonic cultures and from biofilms, before and after antibiotic treatment.

## Materials and methods

### Bacterial strains and culturing conditions

Six bacterial species (clinical strains and one type strain; listed in Table 1) that are frequently co-isolated from CF patients were selected for the development of selective media: *Pseudomonas aeruginosa*, *Staphylococcus aureus*, *Streptococcus anginosus*, *Achromobacter xylosoxidans*, *Rothia mucilaginosa*, and *Gemella haemolysans* [24–27]. For each species, at least one strain was included that was isolated from the respiratory tract or, when this was not readily available in public repositories, a type strain was used.

**Table 1:** Overview of strains used in this study and isolation sites.

Species	Strain	Isolation site
***Pseudomonas aeruginosa***	AA2	Lung; early CF infection ^[^^28^^]^
	AA44	Lung; late CF infection ^[^^28^^]^
	PAO1	Wound
***Staphylococcus aureus***	SP123	Sputum of extubated patient ^[^^29^^]^
	Mu50	Wound
***Streptococcus anginosus***	LMG 14696	Human throat
	LMG 14502	Human respiratory tract
***Achromobacter xylosoxidans***	LMG 26680	Human blood
	LMG 14980	Sputum—Belgian CF patient
***Rothia mucilaginosa***	DSM 20746	Bronchial secretion
	ATCC 49042	Throat
***Gemella haemolysans***	LMG 18984	Unknown
	LMG 1068	Type strain

For all strains, liquid cultures were grown in BHI (Brain Heart Infusion) broth (Lab M) at 37°C with shaking at 250 rpm until stationary phase. For *S*. *anginosus* and *G*. *haemolysans*, cultures were incubated in microaerophilic conditions (±5% O_2_, ±15% CO_2_).

When indicated, microaerophilic conditions (±5% O_2_, ±15% CO_2_) were obtained using the CampyGen Compact system (Oxoid, Thermo Fisher Scientific). To create an anaerobic environment (<1% O_2_), AnaeroGen Compact sachets (Oxoid, Thermo Fischer Scientific) or candle jars with the Anaerocult A system (Merck Millipore) were used.

### General media

For each of the six bacterial species included, a general medium with a theoretical recovery of 100% was used: Luria Bertani (LB; Lab M) agar for *P*. *aeruginosa* and *S*. *aureus*, Brain Heart Infusion (BHI; Lab M) agar for *S*. *anginosus*, Nutrient agar (Lab M) for *A*. *xylosoxidans* and *R*. *mucilaginosa*, and Columbia agar (CA; Lab M) or Columbia Blood Agar (CBA; Columbia Agar base, Lab M + 5% defibrinated sheep blood, Biotrading) for *G*. *haemolysans*.

### Media and chemicals used for development of the selective media

#### Previously described selective media

Previously described selective media were examined for selectivity and adjusted when necessary. For all media, each selective component was examined to distinguish which components were essential for selective inhibition. For *P*. *aeruginosa*, Pseudomonas Isolation Agar (PIA; BD Diagnostics) is described which uses 25 μg/mL triclosan (Irgasan, Sigma Aldrich) as a selective agent since most bacterial species are susceptible while *P*. *aeruginosa* is resistant [30,31]. For *S*. *aureus*, Mannitol Salt Agar is widely used for selection, based on a 7.5% NaCl concentration [32,33]. For *S*. *anginosus*, several media were considered: NAS (nalidixic acid sulfamethazine) agar [19], Mitis Salivarius Agar (Sigma-Aldrich) and Edwards medium (Oxoid, Thermo Fisher Scientific). For *A*. *xylosoxidans*, MCXVAA medium is described [18], based on MacConkey agar supplemented with 5 mg/mL xylose, 20 μg/mL vancomycin, 20 μg/mL aztreonam and 5 μg/mL amphotericin. For *R*. *mucilaginosa*, Rothia Mucilaginosa Selective Medium (RMSM) was previously developed, containing 50 μg/mL sodium selenite and 10 μg/mL colistin [22]. Finally, for *G*. *haemolysans*, a supplemented Edwards medium with 5 μg/mL colistin sulphate and 2.5 μg/mL oxolinic acid was described [20].

#### Antimicrobial susceptibility testing

To identify potential selective or discriminating antibiotic agents, an antibiotic susceptibility screening was performed. Susceptibility of the six species to 25 antibiotics was assessed using the disk diffusion assay (S1 Table). To this end, antibiotic disks were placed on inoculated BHI agar, according to the manufacturer’s instructions. Subsequently, the MIC_90_ of selected antibiotics (S2 Table) was determined using the broth microdilution method according to EUCAST guidelines [34]. For all antibiotics, a range of 0.5 – 256 μg/mL was tested in BHI or nutrient broth, depending on the general medium for the intended strain.

### Selectivity testing

The ability of the medium to select for each of the six species was checked by plating. A planktonic culture of each species, grown to stationary phase in BHI medium, was brought to an OD 590 nm corresponding to approximately 5 x 10^7^ CFU/mL, serially diluted and then plated on the respective general medium as a positive control (theoretical recovery of 100%) and in parallel on each of the candidate selective media (detection limit = 10^2^ CFU/mL). The medium was considered selective if distinct, countable colonies (clear growth) could be observed for the intended species only. All media were incubated at 37°C for 16 hours or until colonies were of sufficient size for counting.

### Formation of single- and multispecies biofilms

All biofilms were grown in a PVC flat-bottomed 96-well microtiter plate (Thermo Fisher) as described previously, with modifications [35,36]. Stationary phase liquid cultures were diluted in BHI supplemented with 2.5% lysed horse blood (horse blood from Biotrading, protocol for lysed horse blood according to EUCAST). For single-species biofilms, 100 μL of a 2.5 x 10^7^ CFU/mL suspension was transferred to a 96-well plate. For multispecies biofilms, 2.5 x 10^6^ CFU of each strain were added per well to obtain an equal number of cells per species per well. The inoculated 96-well plates were incubated at 37°C for 24 hours in a microaerophilic environment, thereby mimicking the low oxygen conditions in the CF lung [37,38].

### Antibiotic treatment of biofilms

After 24 h, biofilms were treated with antibiotics as described previously [35]. Briefly, the biofilms were rinsed with physiological saline solution (0.9% NaCl in milliQ water). Afterwards, 100 μL of medium (BHI + 2.5% lysed horse blood) was added to the control biofilms or 100 μL of antibiotic solution in the same medium was added to the biofilms. Four antimicrobials from four major antibiotic classes were selected. An overview of the antibiotics and their concentrations used is shown in Table 2. As a starting point, one set of antibiotic concentrations that gave a decrease of one to two logs of *P*. *aeruginosa* biofilm was used on all strains. Other concentrations of antibiotics were tested when no effect was observed for the other bacterial species or in case of complete biofilm killing (detection limit of 10^2^ CFU/mL) with the initial test concentration. All biofilms were incubated at 37°C under microaerophilic conditions for an additional 24 hours.

**Table 2:** Overview of antibiotic concentrations used for quantitative recovery experiments.

	Ceftazidime	Ciprofloxacin	Colistin	Tobramycin
***Pseudomonas aeruginosa***	2000 μg/mL	0.5 μg/mL	500 μg/mL	100 μg/mL
***Staphylococcus aureus***	2000 μg/mL	0.5 μg/mL	500 μg/mL	100 μg/mL
		*200* μ*g/mL*		*400* μ*g/mL*
***Streptococcus anginosus***	2000 μg/mL	0.5 μg/mL	500 μg/mL	100 μg/mL
		*4* μ*g/mL*		
***Achromobacter xylosoxidans***	2000 μg/mL	0.5 μg/mL	500 μg/mL	100 μg/mL
	***1000* μ*g/mL***	*4* μ*g/mL*		
***Rothia mucilaginosa***	2000 μg/mL	0.5 μg/mL	500 μg/mL	100 μg/mL
	***1000* μ*g/mL***	*4* μ*g/mL*		
***Gemella haemolysans***	2000 μg/mL	0.5 μg/mL	500 μg/mL	100 μg/mL
		*4* μ*g/mL*		*25* μ*g/mL*

Concentrations in italic show additional concentrations tested when either no effect or complete removal of biofilm growth occurred with the initial concentrations.

### Biofilm quantification and recovery testing

Following antibiotic treatment of single or multispecies biofilms, biofilms were rinsed with physiological saline. To quantify the number of colony forming units, biofilms were homogenized by two rounds of vortexing (900 rpm, 5 min) and sonication (5 min; Branson Ultrasonic bath), as described previously [35]. The resulting suspensions were serially diluted and plated on the appropriate general and/or selective media. Plates were incubated at 37°C overnight (16 hours) or until colonies were of sufficient size for counting. Recovery was considered quantitative when no significant difference was observed between the number of CFU on the general medium compared to the selective medium.

### Statistics

All experiments were carried out at least in biological triplicate. Biofilm experiments contained three technical replicates. Statistical analyses were performed in SPSS 23.0. Normality of the data was checked through the Shapiro-Wilk test. Differences between the means of normally distributed data were assessed by ANOVA-testing, followed by a Dunnett’s post hoc analysis, or by an independent samples t-test. When normality was not confirmed, a Kruskal-Wallis non-parametric test or Mann-Whitney test was performed. Means and standard deviations are shown in each graph. Statistical significance is assumed when p-values are < 0.05.

## Results & discussion

### Assessment of selectivity and quantitative recovery of selective media

**Fig 1:**
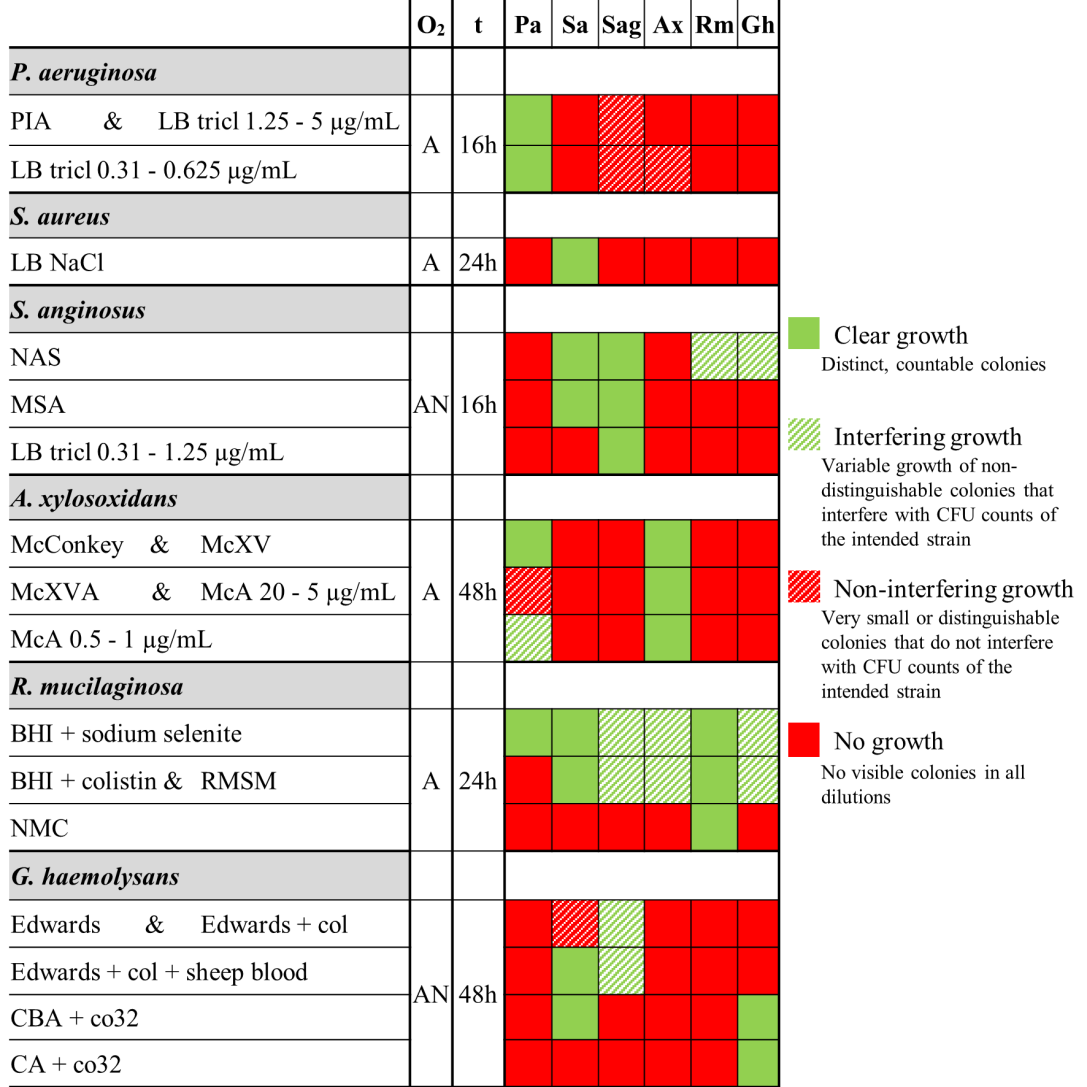
Overview of selectivity experiments. The first column shows the intended species and an abbreviated name of the medium, the second column (O_2_) shows aerobic (A) or anaerobic (AN) incubation, and the third column (t) shows incubation times. Strains belonging to the same species showed the same results. **Pa**: *P*. *aeruginosa* AA2, PAO1, and AA44; **Sa**: *S*. *aureus* SP123 and Mu50; **Sag**: *S*. *anginosus* LMG 14696 and LMG 14502; **Ax**: *A*. *xylosoxidans* LMG 26680 and LMG 14980; **Rm**: *R*. *mucilaginosa* DSM 20746 and ATCC 49042; **Gh**: *G*. *haemolysans* LMG 18984 and LMG 1068. Results are shown for all tested media: Pseudomonas Isolation Agar (**PIA**), LB + triclosan (**LB tricl**), LB + 7.5% NaCl (**LB NaCl**), nalidixic acid sulphamethazine agar (**NAS**), Mitis-salivarius agar (**MSA**), McConkey + 5 mg/mL xylose + 20 μg/mL vancomycin (**McXV**), McConkey + 5 mg/mL xylose + 20 μg/mL vancomycin (**McXV**) + 20 μg/mL aztreonam (**McXVA**), McConkey + aztreonam (**McA**), Rothia mucilaginosa selective medium (RMSM), nutrient agar + 5 μg/mL mupirocin + 10 μg/mL colistin sulphate (**NMC**), Edwards medium + 10 μg/mL colistin sulphate (**Edwards + col**), Columbia blood agar + 32/6.4 μg/mL co-trimoxazole (**CBA + co32**), Columbia agar + 32/6.4 μg/mL co-trimoxazole (**CA + co32**).

**Fig 2:**
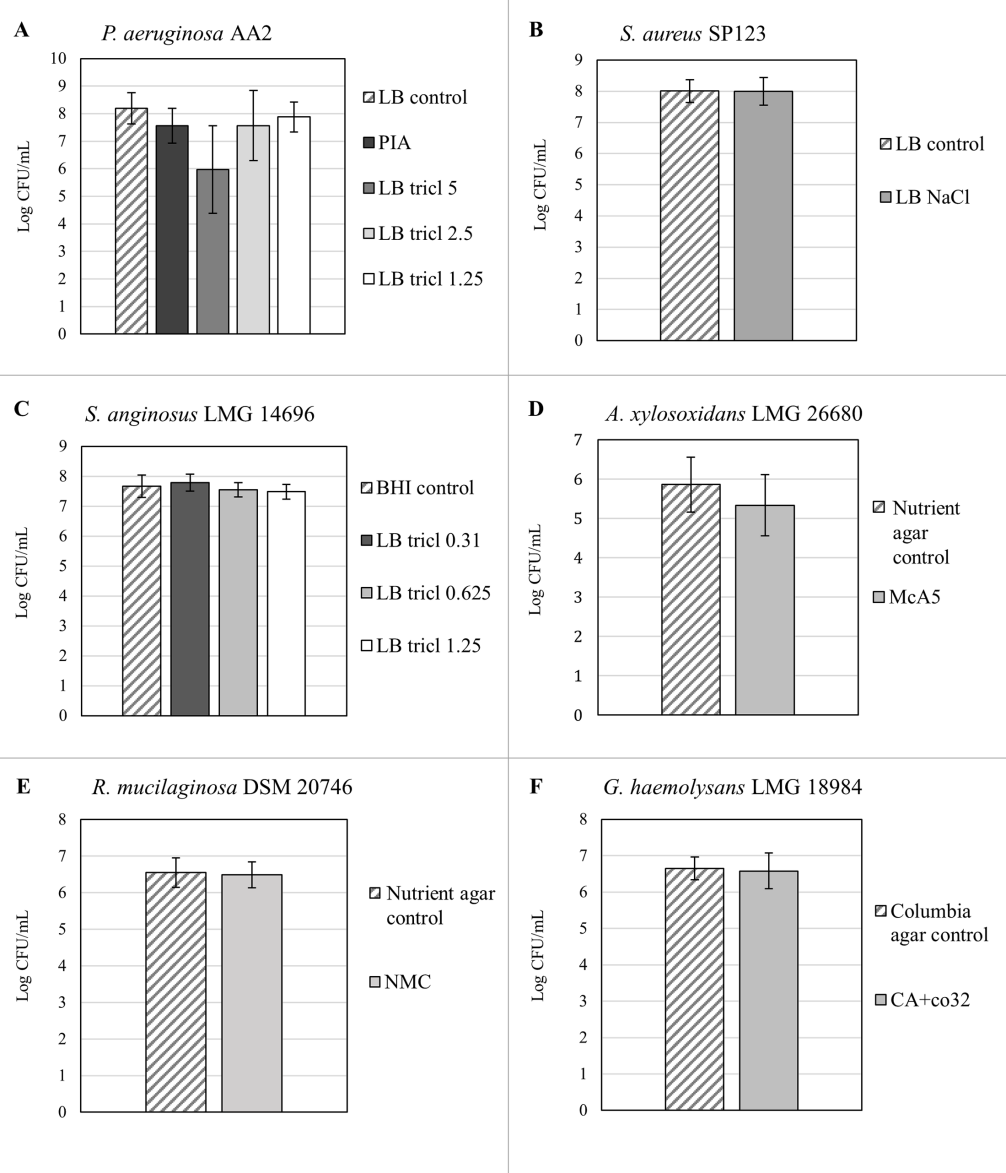
Quantification of untreated biofilms of the six bacterial species on candidate selective media compared to a general medium. **(A)** *P*. *aeruginosa* AA2 **(B)** *S*. *aureus* SP 123 **(C)** *S*. *anginosus* LMG 14696 **(D)** *A*. *xylosoxidans* LMG 26680 **(E)** *R*. *mucilaginosa* DSM 20476 **(F)** *G*. *haemolysans* LMG 18984. PIA = Pseudomonas Isolation Agar; LB tricl = LB agar supplemented with triclosan in various concentrations (μg/mL); LB NaCl = LB supplemented with 7.5% NaCl; McA5 = McConkey agar supplemented with 5 μg/mL aztreonam; NMC = nutrient agar supplemented with 5 μg/mL mupirocin and 10 μg/mL colistin sulphate; CA + co32 = Columbia agar with 32/6.4 μg/mL co-trimoxazole. Graphs show mean recovery and standard deviations. * p < 0.05, n ≥ 3.

The results of the selectivity testing using planktonic cultures on previously described and modified selective media are summarized in Fig 1. Following a positive outcome for selectivity, the selective media were evaluated for quantitative recovery of cells from untreated biofilms (Fig 2).

#### P. aeruginosa

We confirmed selectivity of Pseudomonas Isolation Agar (PIA, containing 25 μg/mL triclosan). In order to reduce the concentration of the selective agent, lower triclosan concentrations (5 – 0.31 μg/mL in LB agar) were tested. All tested concentrations of triclosan inhibited growth of all the other bacteria while allowing growth of *P*. *aeruginosa* after aerobic overnight (16h) incubation. At concentrations of 1.25 μg/mL triclosan or higher, *S*. *anginosus* showed growth of small colonies after 16h incubation which did not interfere with quantification of *P*. *aeruginosa*. It is important to notice that longer incubation times (24-48h) lead to interference of *S*. *anginosus* and *A*. *xylosoxidans* growth for quantification of *P*. *aeruginosa*, highlighting the importance of selecting appropriate incubation times. Subsequently, recovery of a single-species biofilm was tested on triclosan-supplemented LB agar. Addition of 5 μg/mL triclosan resulted in a significant decrease of *P*. *aeruginosa* recovery (p < 0.05), while addition of 2.5 and 1.25 μg/mL triclosan did not affect recovery. Hence, LB agar with 1.25 μg/mL triclosan (LB tricl 1.25) was used for further experiments.

#### S. aureus

Mannitol Salt Agar contains 7.5% NaCl, and we evaluated LB agar supplemented with the same amount of NaCl (LB NaCl). Aerobic overnight incubation showed that only *S*. *aureus* was able to grow on this medium, confirming its selectivity. Furthermore, recovery of biofilm cells on LB NaCl showed no difference in CFU counts compared to LB agar.

#### S. anginosus

NAS agar and Mitis Salivarius Agar combined with anaerobic incubation were examined for selectivity but both media also supported growth of *S*. *aureus* and *R*. *mucilaginosa* in these conditions. Since 1.25 μg/mL triclosan permitted growth of *S*. *anginosus*, LB supplemented with 1.25, 0.625 and 0.31 μg/mL triclosan was tested. Incubation took place under anaerobic conditions to inhibit *P*. *aeruginosa* growth, as *P*. *aeruginosa* cannot grow anaerobically without an alternative terminal electron acceptor [39,40]. This approach allowed growth of *S*. *anginosus* only. In addition, LB with triclosan (LB 1.25, 0.625, 0.31) also allowed quantitative recovery of cells from single-species *S*. *anginosus* biofilms. Based on these results, LB tricl 0.31 (with anaerobic incubation) was selected for further experiments.

#### A. xylosoxidans

MCXVAA (based on MacConkey agar supplemented with 5 μg/mL xylose, 20 μg/mL vancomycin, 20 μg/mL aztreonam and 5 μg/mL amphotericin) [18], was confirmed to be a selective medium for *A*. *xylosoxidans*. The selective value of each of these components (except for the anti-fungal amphotericin) was subsequently tested. MacConkey agar inhibited growth of the Gram-positive bacteria, while addition of 20 μg/mL aztreonam inhibited growth of *P*. *aeruginosa*. MacConkey agar with 1 μg/ml (or less) aztreonam also supported growth of *P*. *aeruginosa*, while at concentrations of 10 μg/mL or higher, partial growth inhibition of *A*. *xylosoxidans* was observed. MacConkey supplemented with 5 μg/mL aztreonam (McA5) was tested further and this medium allowed quantitative recovery of cells from untreated *A*. *xylosoxidans* single-species biofilms.

#### R. mucilaginosa

RMSM only inhibited growth of *P*. *aeruginosa*, while the other five species, including *R*. *mucilaginosa*, showed at least partial growth on this medium. Growth inhibition of *P*. *aeruginosa* was due to 10 μg/mL colistin sulphate, while sodium selenite did not inhibit growth of any of the species investigated. Results from disk diffusion assays suggested that mupirocin could be a suitable selective agent (S1 Table). Based on MIC values (S2 Table), 5 μg/mL mupirocin was added to nutrient agar, together with 10 μg/mL colistin sulphate. This medium (NMC) consisting of nutrient agar, mupirocin and colistin sulphate, was selective for *R*. *mucilaginosa* and allowed quantitative recovery of *R*. *mucilaginosa* from untreated biofilms.

#### G. haemolysans

We could not confirm selectivity for the previously described supplemented Edwards medium [20] as it did not allow growth of *G*. *haemolysans* in our experiments. To develop a new selective medium for *G*. *haemolysans*, co-trimoxazole was used as this antibiotic did not inhibit *G*. *haemolysans* growth in the disk diffusion assay, while inhibition zones were observed for all other species (S1 Table). Based on MIC values (S2 Table) for all strains, 32/6.4 μg/mL co-trimoxazole was added to CBA. However, besides *G*. *haemolysans*, *S*. *aureus* also showed growth on CBA agar with co-trimoxazole. Yet, in the absence of blood complete growth inhibition was observed for all bacteria except for *G*. *haemolysans*, which lead to the use of Columbia agar with 32/6.4 μg/mL co-trimoxazole (CA + co32) for further testing. Our data showed that the CA + co32 medium allowed quantitative recovery of *G*. *haemolysans* from untreated biofilms.

### Quantitative recovery of biofilm cells after antibiotic treatment

**Fig 3:**
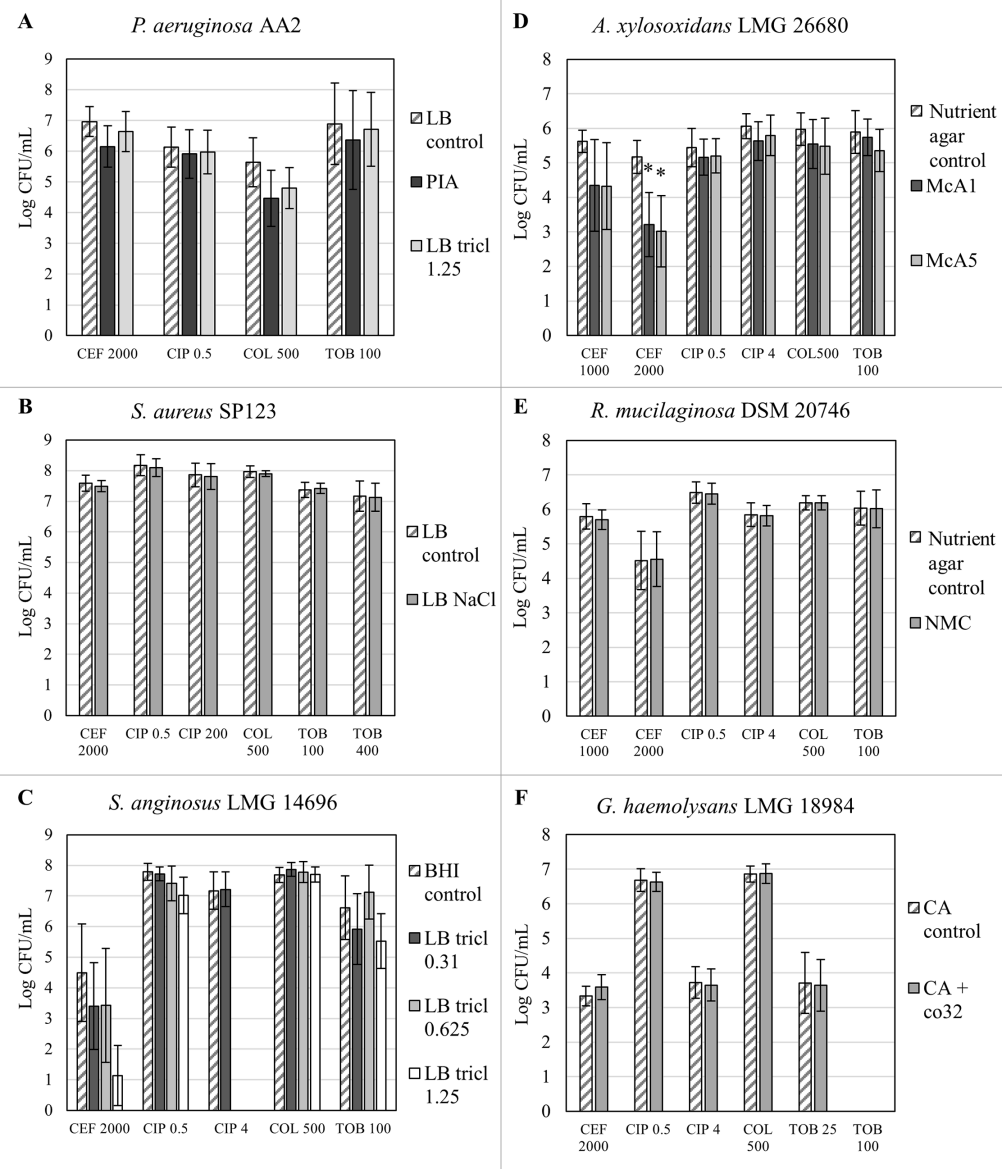
Quantification of biofilms after antibiotic treatment on selective media compared to a general medium. **(A)** *P*. *aeruginosa* AA2 **(B)** *S*. *aureus* SP 123 **(C)** *S*. *anginosus* LMG 14696 **(D)** *A*. *xylosoxidans* LMG 26680 **(E)** *R*. *mucilaginosa* DSM 20476 **(F)** *G*. *haemolysans* LMG 18984. PIA = Pseudomonas Isolation Agar; LB tricl = LB agar supplemented with triclosan in various concentrations (μg/mL); LB NaCl = LB supplemented with 7.5% NaCl; McA5 = McConkey agar supplemented with 5 μg/mL aztreonam; NMC = nutrient agar supplemented with 5 μg/mL mupirocin and 10 μg/mL colistin sulphate; CA + co32 = Columbia agar with 32/6.4 μg/mL co-trimoxazole. Graphs show mean recovery and standard deviations. * p < 0.05, n ≥ 3.

Treated biofilms were plated on the developed selective media and on general media to assess quantitative recovery following antibiotic treatment. Results are summarised in Fig 3. The optimized selective media are presented in Table 3.

**Table 3:** Composition of optimized selective media.

	Selective medium	Composition
***Pseudomonas aeruginosa***	LB tricl 1.25	LB agar
		1.25 μg/mL triclosan
***Staphylococcus aureus***	LB NaCl	LB agar
		7.5% NaCl
***Streptococcus anginosus***	LB tricl 0.31 (anaerobic)	LB agar
		0.31 μg/mL triclosan
***Achromobacter xylosoxidans***	McA5	McConkey agar
		5 μg/mL aztreonam
***Rothia mucilaginosa***	NMC	Nutrient agar
		5 μg/mL mupirocin
		10 μg/mL colistin sulphate
***Gemella haemolysans***	CA + co32	Columbia agar
		32/6.4 μg/mL co-trimoxazole

For *P*. *aeruginosa*, no significant difference was observed between the recovery of biofilm cells on LB, LB with 1.25 μg/mL triclosan, or PIA after treatment with ceftazidime, ciprofloxacin, colistin or tobramycin. Thus, LB tricl 1.25 with aerobic overnight incubation was an appropriate medium for quantitative recovery of *P*. *aeruginosa* biofilm cells after antibiotic treatment.

Similarly, in terms of recovery of *S*. *aureus* biofilms cells, there was no significant difference between LB and LB + 7.5% NaCl after treatment with ceftazidime, ciprofloxacin, colistin or tobramycin.

After treatment with ceftazidime, ciprofloxacin, colistin, or tobramycin, complete recovery of *S*. *anginosus* biofilm cells was observed on LB supplemented with 1.25 μg/mL, 0.625 μg/mL or 0.31 μg/mL triclosan (incubated anaerobically) as no differences with recovery on BHI were observed. For further experiments LB with 0.31 μg/ml triclosan (LB tricl 0.31) was used together with anaerobic incubation.

On McConkey agar with 5 μg/mL aztreonam, no significant difference in *A*. *xylosoxidans* recovery was observed after treatment with ciprofloxacin, colistin or tobramycin, compared to nutrient agar. However, after treatment with 2000 μg/mL ceftazidime, a significant decrease (p < 0.05) in recovery on McA5 was noted compared to nutrient agar. Hence, a lower concentration of aztreonam in MacConkey agar (1 μg/mL; McA1) was tested but this did not restore recovery of the *A*. *xylosoxidans* biofilm treated with ceftazidime. To test whether this effect was dependent on the concentration of the antibiotic, the concentration of ceftazidime was lowered to 1000 μg/mL, which did improve biofilm recovery on both tested media (McA1 and McA5). However, as seen in the previous selectivity experiments, McA1 showed suboptimal inhibition of *P*. *aeruginosa* and furthermore did not lead to increased recovery compared to McA5. Taken together, McA5 was used as a selective medium for *A*. *xylosoxidans*. However, it remains important to keep in mind that recovery on McA5 is affected when biofilms are treated with ceftazidime, which varies dependent on the ceftazidime concentration. It is therefore important to consider that highly concentrated supplementations to selective media may strongly influence quantitative recovery, in particular after preceding antibiotic treatment.

For *R*. *mucilaginosa*, no significant difference between the recovery on NMC and nutrient agar was observed after treatment with either ceftazidime, ciprofloxacin, colistin or tobramycin. The NMC medium was used for quantitative recovery of *R*. *mucilaginosa* following antibiotic treatment in further experiments.

After treatment with ceftazidime, ciprofloxacin, colistin or tobramycin, no significant difference in recovery of *G*. *haemolysans* on the developed selective medium (CA + co32) compared to Columbia agar was observed, confirming the developed selective medium for quantification after antibiotic treatment.

### Validation of the selective media

For further validation of the six novel selective media, an additional strain (another clinical isolate or type strain) of each species was tested (S1 and S2 Figs). The developed selective media allowed quantitative recovery of all the additional strains, with the exception of *A*. *xylosoxidans* LMG 14980 after ceftazidime treatment, and *G*. *haemolysans* LMG 1068 for both the untreated and treated (with ceftazidime and ciprofloxacin) single-species biofilm. Furthermore, a mixed planktonic culture with a defined composition of the six bacteria (± 5 x 10^7^ CFU/mL of each) was plated on the six selective media and recovery was compared to the recovery of single-species cultures. The results show no significant decrease in recovery of the strains in the mixed culture compared to the single-species cultures showing that no growth inhibition occurred and that the mixed culture did not decrease recovery of the individual strains (S3 Fig).

### Proof of concept: Multispecies biofilms

**Fig 4:**
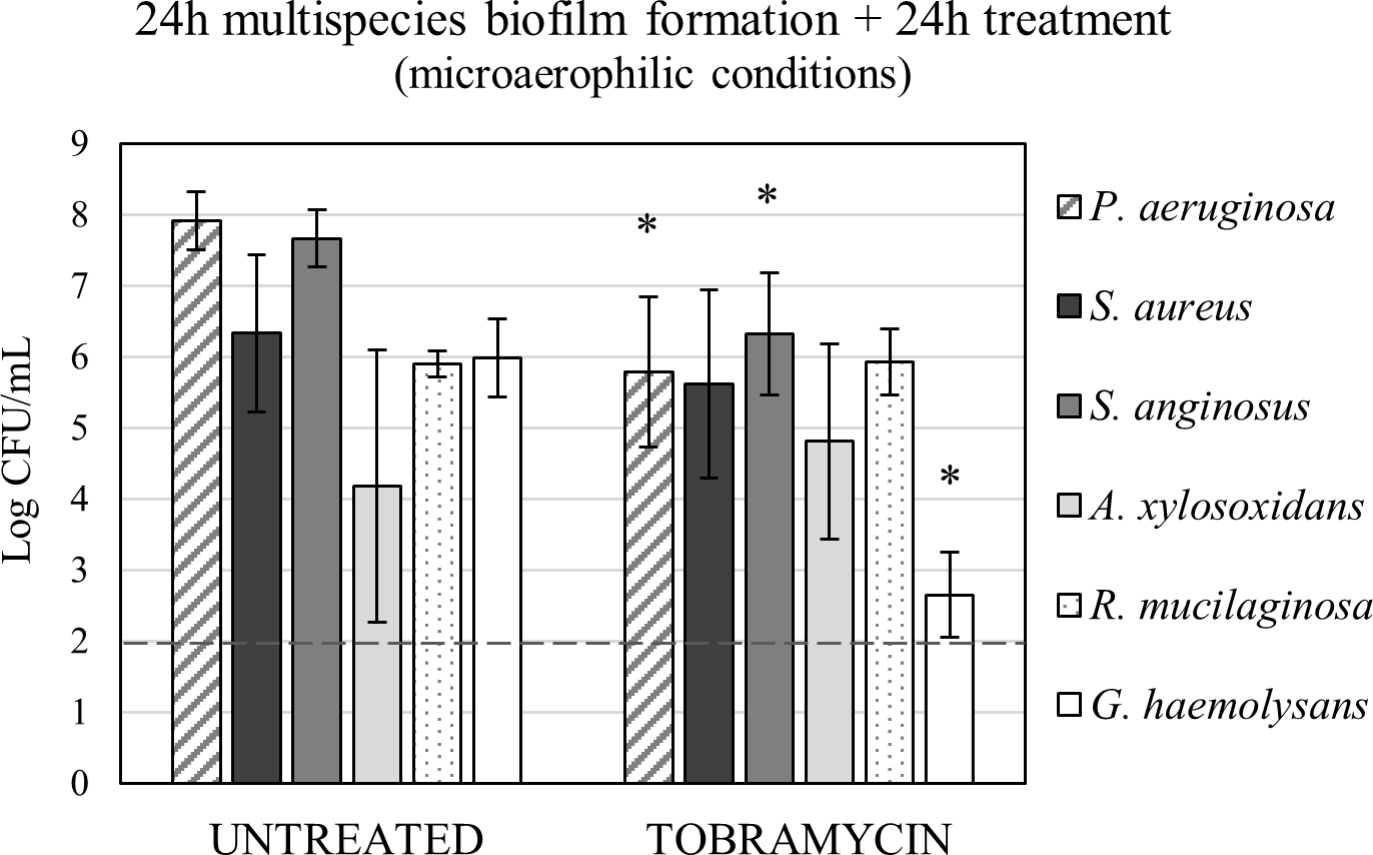
Proof of concept: Quantification of multispecies biofilms. A multispecies biofilm of the six bacterial species was grown for 24 hours and subsequently incubated with fresh medium as an untreated control or treated with 100 μg/mL tobramycin in medium for an additional 24 hours in microaerophilic conditions. Graphs show mean recovery and standard deviations. The detection limit of 10^2^ CFU/mL is represented by a dashed line. * p < 0.05, n ≥ 3.

Finally, as a proof of concept experiment to show that the media can be used to isolate strains grown in a biofilm, the six bacterial species were quantified in multispecies biofilms, before and after treatment with 100 μg/mL tobramycin. Biofilms were grown for 24h and subsequently treated for 24h in microaerophilic conditions (Fig 4). The results indicate that in the multispecies biofilm *P*. *aeruginosa*, *S*. *anginosus* and *G*. *haemolysans* become less abundant after treatment with tobramycin (p < 0.05), while *S*. *aureus*, *A*. *xylosoxidans* and *R*. *mucilaginosa* are not affected by the treatment. Furthermore, biofilms were also grown for 48h and subsequently treated for 24h (S4 Fig). All species, except for *G*. *haemolysans*, could be isolated from this multispecies biofilm before and after treatment as well. These findings show that community composition can be altered by antibiotic treatment. Hence, deciphering the dynamics of multispecies consortia in response to antibiotic treatment using *in vitro* tools may help improve our understanding of how multispecies communities respond to antibiotics *in vivo*.

## Conclusion

In this study, novel selective media were developed that allow quantitative recovery of (un)treated multispecies biofilms comprised of six bacteria frequently co-isolated from CF samples. We observed that incubation conditions, including incubation time and oxygen concentration, were essential to enable selective recovery. Furthermore, evaluation of the developed selective media for quantitative recovery of biofilms before and after antibiotic treatment revealed that certain selective agents profoundly affect recovery. These findings highlight the importance of keeping antibiotic supplementation in selective media to a minimum and to evaluate and potentially modify available selective media to ensure optimal recovery. In conclusion, six selective media were optimized for multispecies biofilm recovery, and we demonstrated that if a selective medium is to be used for quantitative recovery of untreated or antibiotic-treated cultures, prior recovery assessment is indispensable to avoid introducing biases for data interpretation.

## Supporting information

S1 Table Antibiotic disk diffusion assay. Mean inhibition zones and standard deviations (SD) are shown in mm per strain, ‘z’ shows that a clearer zone could be observed, yet no complete inhibition occurred. This assay was performed for *P*. *aeruginosa* PAO1, AA2, and AA44; *S*. *aureus* SP123; *S*. *anginosus* LMG 14696; *A*. *xylosoxidans* LMG 26680; *R*. *mucilaginosa* DSM 20746; *G*. *haemolysans* LMG 18984.
(PDF)

S2 Table MIC data overview. Minimal Inhibitory Concentrations (range of 256–0.5 μg/mL) were determined for gentamicin and mupirocin in nutrient broth, and for co-trimoxazole (SMT-TMP) in BHI broth. -, not determined.
(PDF)

S1 Fig Quantification of untreated (left) and treated (right) biofilms of additional strains. **(A)** *P*. *aeruginosa* PAO1 and AA44, **(B)** *S*. *aureus* Mu50, PIA = Pseudomonas Isolation Agar; LB tricl = LB agar supplemented with triclosan in various concentrations (μg/mL); LB NaCl = LB supplemented with 7.5% NaCl. Graphs show mean recovery and standard deviations. * p < 0.05, n ≥ 3.
(TIF)

S2 Fig Quantification of untreated (left) and treated (right) biofilms of additional strains. **(C)** *S*. *anginosus* LMG 14502, **(D)** *A*. *xylosoxidans* LMG 26680, **(E)** *R*. *mucilaginosa* ATCC 49042, and **(F)** *G*. *haemolysans* LMG 18984. LB tricl = LB agar supplemented with triclosan in various concentrations (μg/mL); McA5 = McConkey agar supplemented with 5 μg/mL aztreonam; NMC = nutrient agar supplemented with 5 μg/mL mupirocin and 10 μg/mL colistin sulphate; CA + co32 = Columbia agar with 32/6.4 μg/mL co-trimoxazole. Graphs show mean recovery and standard deviations. * p < 0.05, n ≥ 3.
(TIF)

S3 Fig Validation of the novel selective media. Planktonic mono-species cultures and a mixed culture were plated on the newly developed selective media and showed equal recovery. Graphs show mean recovery and standard deviations.
(TIF)

S4 Fig Proof of concept: Quantification of multispecies biofilms. A multispecies biofilm of the six bacterial species was grown for 48 hours and subsequently incubated with fresh medium as an untreated control or treated with 100 μg/mL tobramycin in medium for an additional 24 hours in microaerophilic conditions. Graphs show mean recovery and standard deviations. The detection limit of 10^2^ CFU/mL is represented by a dashed line. * p < 0.05, n ≥ 3.
(TIF)
