# Supplementary material for: Developing selective media for quantification of multispecies biofilms following antibiotic treatment
Source: PLoS One. 2017 Nov 9;12(11):e0187540. doi: 10.1371/journal.pone.0187540 (PMC5679531; doi:10.1371/journal.pone.0187540)
Supplement: S1 Table — Mean inhibition zones and standard deviations (SD) are shown in mm per strain, ‘z’ shows that a clearer zone could be observed, yet no complete inhibition occurred. This assay was performed for P. aeruginosa PAO1, AA2, and AA44; S. aureus SP123; S. anginosus LMG 14696; A. xylosoxidans LMG 26680; R. mucilaginosa DSM 20746; G. haemolysans LMG 18984. (PDF) [file pone.0187540.s001.pdf]

S1 Table. Antibiotic disk diffusion assay.

		P. aeruginosa						*S. aureus*		*S. anginosus*		*A. xylosoxidans*		*R. mucilaginosa*		*G. haemolysans*	
AB	*Conc. (μg)*	PAO1	SD	AA2	SD	AA44	SD	SP123	SD	LMG 14696	SD	DSM 20746	SD	LMG 26680	SD	LMG 18984	SD
CAZ	30	10	1	10	0	10	2	z		9	1	9	1	12	0	15	4
CL	10	4	1	3	0	4	0	0	0	0	0	0	0	7	0	1	1
FEP	30	11	1	8	1	9	0	1	1	11	1	10	4	4	1	19	4
OFX	5	9	1	11	1	7	0	2	0	7	1	7	0	7	1	9	1
RD	5	2	1	1	0	1	0	12	1	13	0	11	1	0	0	12	0
AMP	2	0	0	0	0	0	0	0	0	13	1	12	1	0	0	19	3
VA	30	0	0	0	0	0	0	4	0	7	1	6	1	0	0	11	2
SXT	25	3		7	1	0	0	10	1	12	1	7	1	4	1	0	0
KZ	30	0	0	0	0	0	0	1	0	12	2	13	3	0	0	17	5
OX	5	0	0	0	0	0	0	0		11	1	10	4	0	0	16	3
ATM	30	9	1	13	1	6	6	0	0	0	0	4	1	0	0	9	2
NN	10	8	1	8	1	9	1	6	1	0	0	1	0	0	0	5	0
PB	300 IU	5	1	5	0	6	1	1		0	0	2	1	10	0	1	1
LVX	5	10	1	11	1	7	0	4	1	7	0	7	1	10	1	11	1
B	10 IU	0	0	0	0	0	0	5	0	7	1	11	1	0	0	10	2
MEM	10	0	0	0	0	0	0	0	0	2	1	5	4	0	0	7	4
CIP	5	15	0	13	0	12	0	2	1	8	1	7	1	11	1	10	3
NB	30	0	0	2	1	0		8	1	8	2	7	2	9	5	14	6
E	15	4	1	3	0	1		0	0	11	1	12	1	0		17	5
CRO	30	5	3	z		4	4	z		12	1	9	3	3	1	21	4
IPM	10	9	1	7	0	10	1	2	0	15	3	11	4	9	3	23	4
DA	10	0	0	0	0	0	0	0		9	2	7	1	0	0	10	4
TE	30	6	0	6	0	7	1	10	1	11	2	11	1	15	2	15	6
CN	10	8	1	7	1	8	1	6	1	2	0	5	0	0	0	6	3
MUP	5	0	0	1	0	0	0	13	1	13	2	2	2	14	1	19	6

Mean inhibition zones and standard deviations (SD) are shown in mm per strain, 'z' shows that a clearer zone could be observed, yet no complete inhibition occurred. This assay was performed for *P. aeruginosa* PAO1, AA2, and AA44; *S. aureus* SP123; *S. anginosus* LMG 14696; *A. xylosoxidans* LMG 26680; *R. mucilaginosa* DSM 20746; *G. haemolysans* LMG 18984. Antibiotic disks (AB) and concentrations (Conc. μg) are shown in column 1 and 2. Disk abbreviations are listed below.

CAZ	ceftazidime	OX	oxacillin	NB	novobiocin
CL	colistin	ATM	aztreonam	E	erythromycin
FEP	cefepime	NN	tobramycin	CRO	ceftriaxone
OFX	ofloxacin	PB	polymyxin B	IPM	imipenem
RD	rifampicin	LVX	levofloxacin	DA	clindamycin
AMP	ampicillin	B	bacitracin	TE	tetracyclin
VA	vancomycin	MEM	meropenem	CN	gentamycin
SXT	sulphametoxazole/trimethoprim	CIP	ciprofloxacin	MUP	mupirocin
KZ	cephazolin				
