# Supplementary material for: Developing selective media for quantification of multispecies biofilms following antibiotic treatment
Source: PLoS One. 2017 Nov 9;12(11):e0187540. doi: 10.1371/journal.pone.0187540 (PMC5679531; doi:10.1371/journal.pone.0187540)
Supplement: S2 Table — Minimal Inhibitory Concentrations (range of 256–0.5 μg/mL) were determined for gentamicin and mupirocin in nutrient broth, and for co-trimoxazole (SMT-TMP) in BHI broth. -, not determined. (PDF) [file pone.0187540.s002.pdf]

S2 Table. MIC data overview.

	MUPIROCIN	SMT-TMP
**PAO1**	>256 µg/mL	32 µg/mL
**AA2**	>256 µg/mL	8 µg/mL
**AA44**	> 256 µg/mL	-
**SP123**	< 0.5 µg/mL	8 µg/mL
**LMG 14696**	1 µg/mL	< 0.5 µg/mL
**LMG 26680**	4 µg/mL	< 0.5 µg/mL
**DSM 20476**	>256 µg/mL	32 µg/mL
**LMG 18984**	< 0.5 µg/mL	> 256 µg/mL

Minimal Inhibitory Concentrations (range of 256 – 0.5 µg/mL) were determined for mupirocin in nutrient broth, and for co-trimoxazole (SMT-TMP) in BHI broth. -, not determined.
