# Supplementary figures and images for: Developing selective media for quantification of multispecies biofilms following antibiotic treatment
Source: PLoS One. 2017 Nov 9;12(11):e0187540. doi: 10.1371/journal.pone.0187540 (PMC5679531; doi:10.1371/journal.pone.0187540)

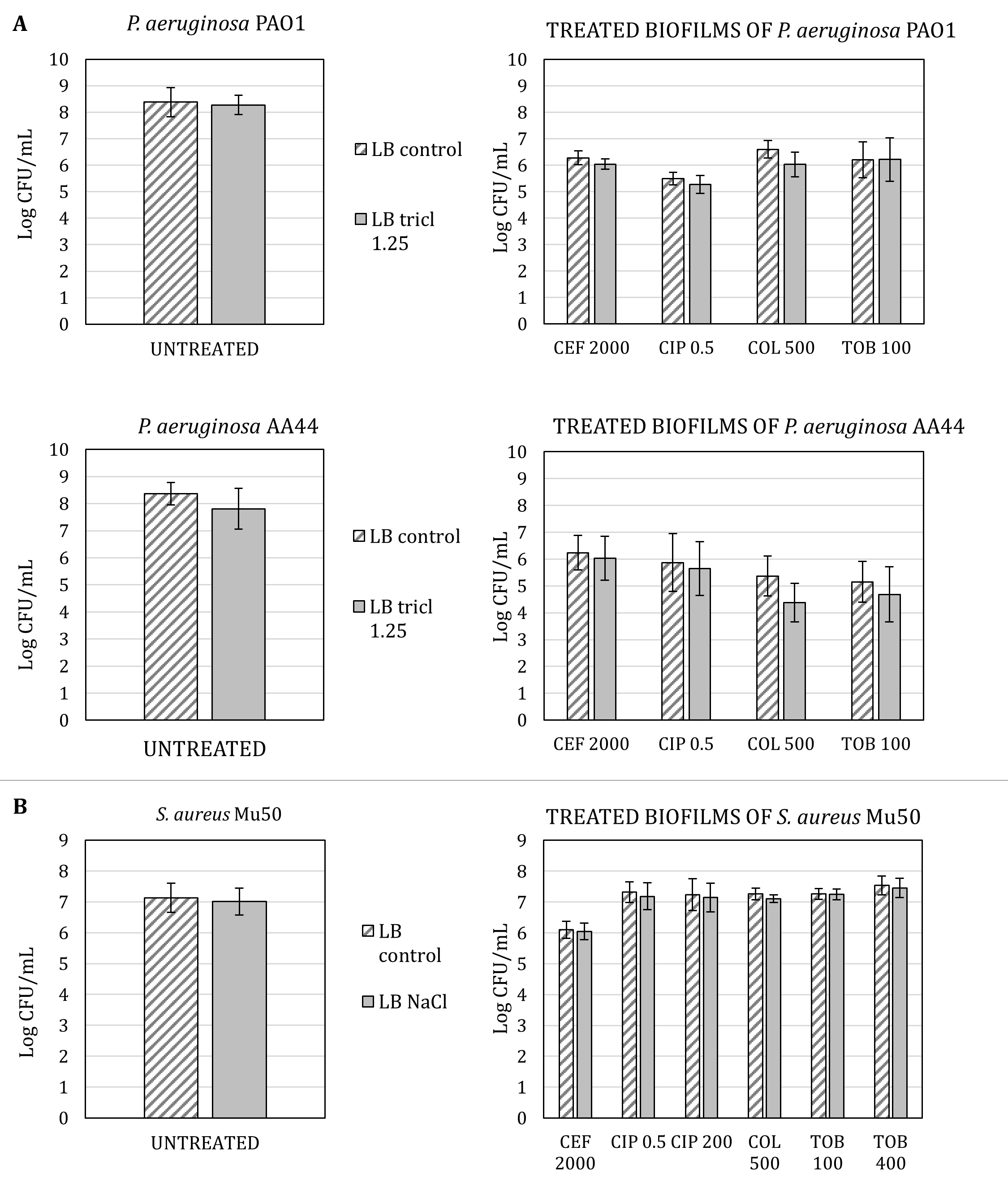

Supplement: S1 Fig — (A) P. aeruginosa PAO1 and AA44, (B) S. aureus Mu50, PIA = Pseudomonas Isolation Agar; LB tricl = LB agar supplemented with triclosan in various concentrations (μg/mL); LB NaCl = LB supplemented with 7.5% NaCl. Graphs show mean recovery and standard deviations. * p < 0.05, n ≥ 3. (TIF) [file pone.0187540.s003.tif]

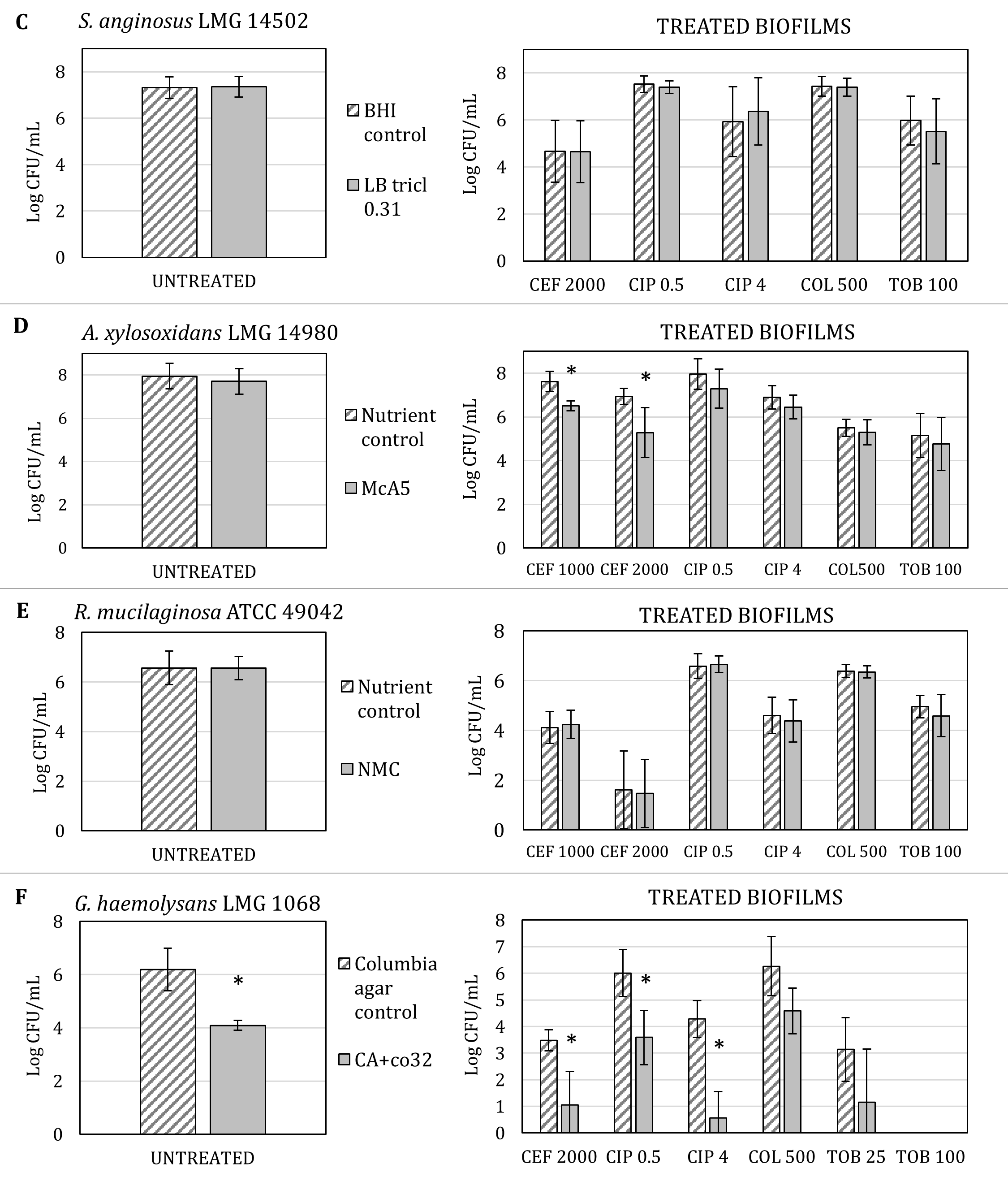

Supplement: S2 Fig — (C) S. anginosus LMG 14502, (D) A. xylosoxidans LMG 26680, (E) R. mucilaginosa ATCC 49042, and (F) G. haemolysans LMG 18984. LB tricl = LB agar supplemented with triclosan in various concentrations (μg/mL); McA5 = McConkey agar supplemented with 5 μg/mL aztreonam; NMC = nutrient agar supplemented with 5 μg/mL mupirocin and 10 μg/mL colistin sulphate; CA + co32 = Columbia agar with 32/6.4 μg/mL co-trimoxazole. Graphs show mean recovery and standard deviations. * p < 0.05, n ≥ 3. (TIF) [file pone.0187540.s004.tif]

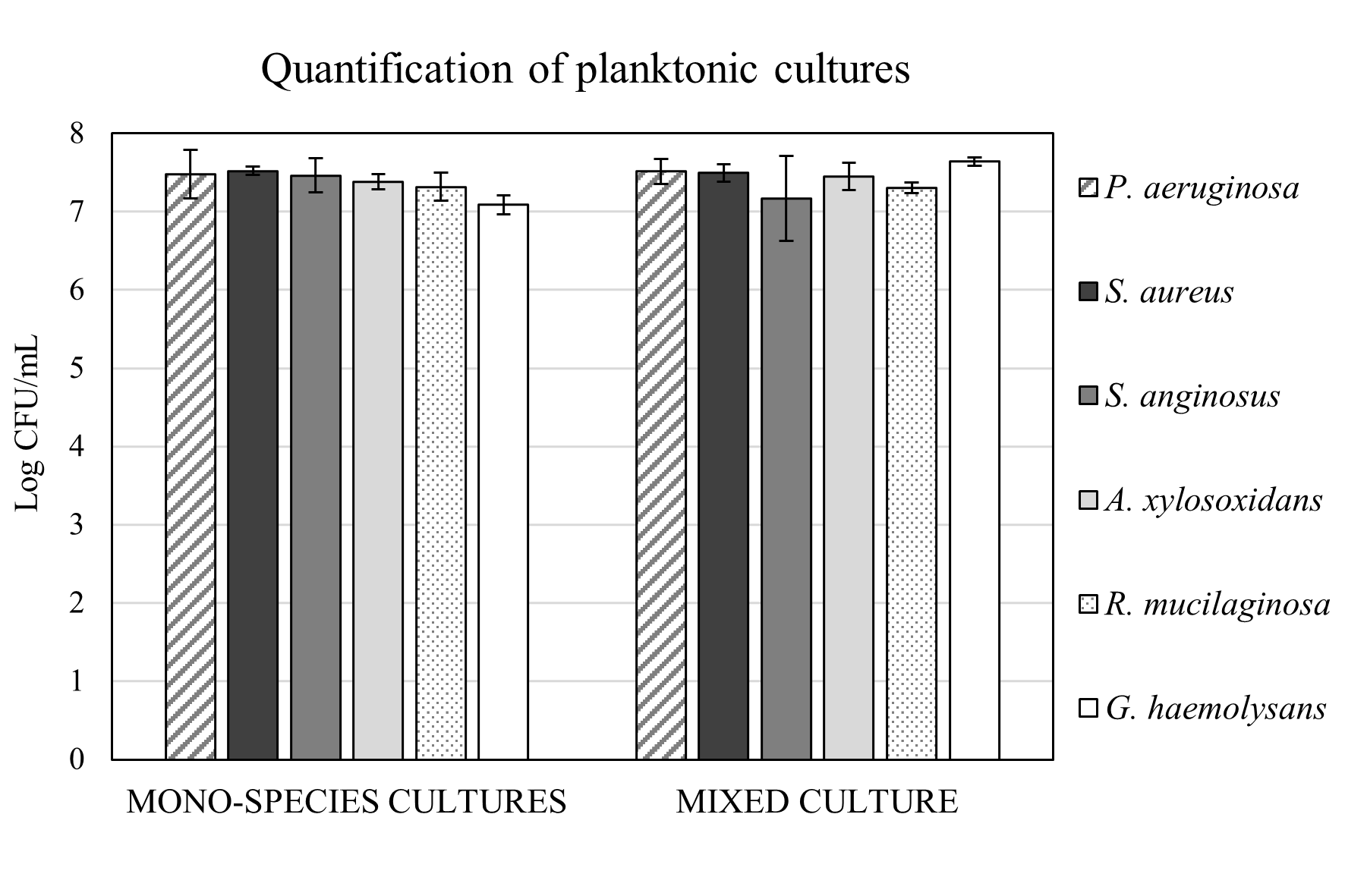

Supplement: S3 Fig — Planktonic mono-species cultures and a mixed culture were plated on the newly developed selective media and showed equal recovery. Graphs show mean recovery and standard deviations. (TIF) [file pone.0187540.s005.tif]

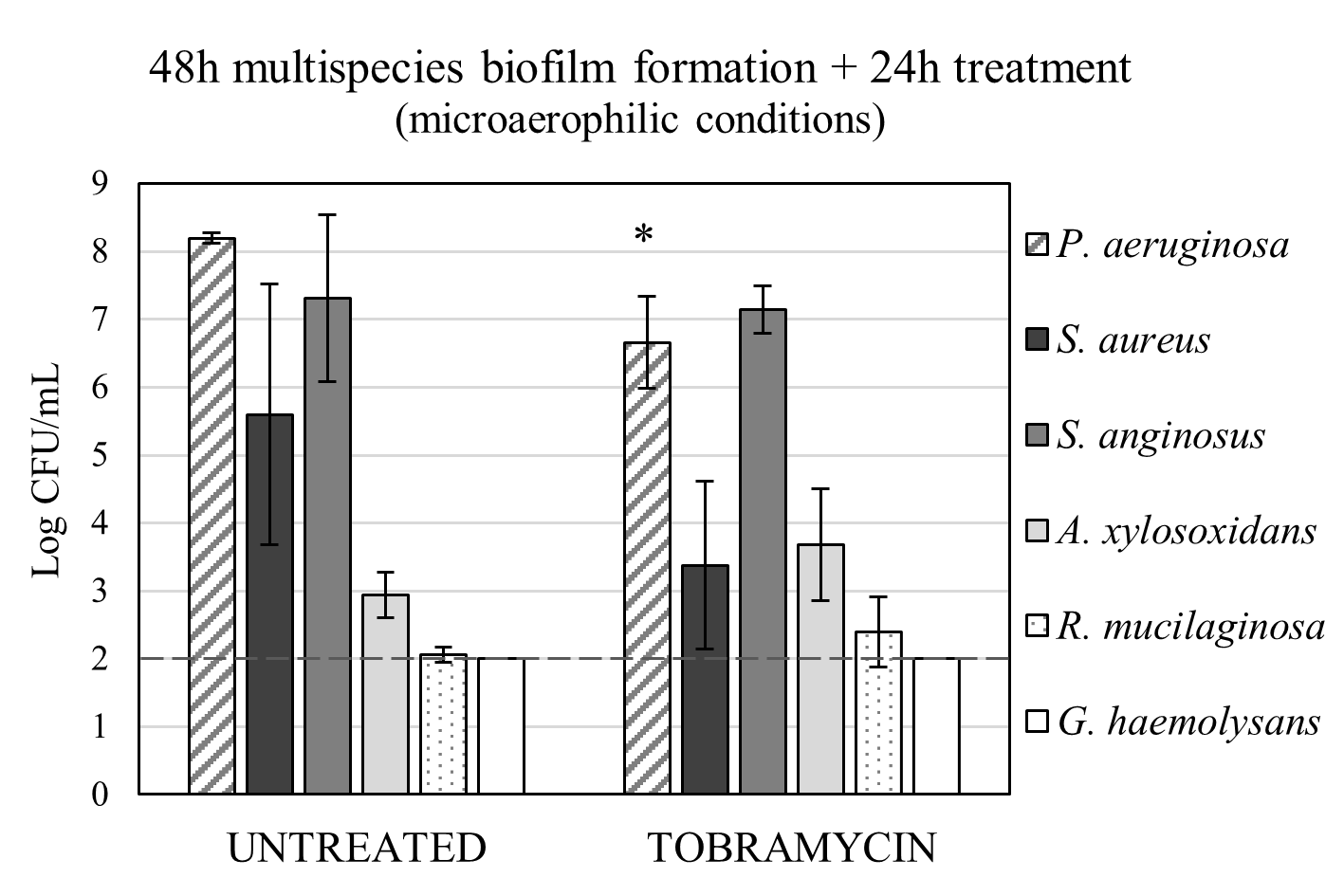

Supplement: S4 Fig — A multispecies biofilm of the six bacterial species was grown for 48 hours and subsequently incubated with fresh medium as an untreated control or treated with 100 μg/mL tobramycin in medium for an additional 24 hours in microaerophilic conditions. Graphs show mean recovery and standard deviations. The detection limit of 102 CFU/mL is represented by a dashed line. * p < 0.05, n ≥ 3. (TIF) [file pone.0187540.s006.tif]
